# A Subset-Reduced Method for FDE ARAIM of Tightly-Coupled GNSS/INS

**DOI:** 10.3390/s19224847

**Published:** 2019-11-07

**Authors:** Weichuan Pan, Xingqun Zhan, Xin Zhang, Shizhuang Wang

**Affiliations:** School of Aeronautics and Astronautics, Shanghai Jiao Tong University, Shanghai 200240, China; panweic@sjtu.edu.cn (W.P.); sz.wang@sjtu.edu.cn (S.W.)

**Keywords:** ARAIM, GNSS/INS, BDS, integrity

## Abstract

The advanced receiver autonomous integrity monitoring (advanced RAIM, ARAIM) is the next generation of RAIM which is widely used in civil aviation. However, the current ARAIM needs to evaluate hundreds of subsets, which results in huge computational loads. In this paper, a method using the subset excluding entire constellation to evaluate the single satellite fault subsets and the simultaneous multiple satellites fault subsets is presented. The proposed ARAIM algorithm is based on the tight integration of the global navigation satellite system (GNSS) and inertial navigation system (INS). The number of subsets that the proposed GNSS/INS ARAIM needs to consider is about 2% of that of the current ARAIM, which reduces the computational load dramatically. The detailed fault detection (FD) process and fault exclusion (FE) process of the proposed GNSS/INS ARAIM are provided. Meanwhile, the method to obtain the FD-only integrity bound and the after-exclusion integrity bound is also presented in this paper. The simulation results show that the proposed GNSS/INS ARAIM is able to find the failing satellite accurately and its integrity performance is able to meet the integrity requirements of CAT-I precision approach.

## 1. Introduction

With the development of civil aviation, the number of aircraft in the same airspace increases greatly. Therefore, there is an increasing demand to improve flight efficiency. The global navigation satellite system (GNSS) is very important for flight efficiency improvement. However, the application of GNSS must be able to meet the stringent navigation performance requirements of civil aviation. The International Civil Aviation Organization (ICAO) specifies the navigation performance in terms of integrity, accuracy, continuity, and availability [1]. Integrity reflects correctness of a position solution and it is the ability of a navigation system to alert the users timely when the position errors exceeding specified requirements [2]. Integrity monitoring is very important to safeguard the safety of the crew and passengers [3]. Continuity is the ability of a navigation system to maintain the required performance. Both integrity and continuity are important for civil aviation to safeguard safety-of-life (SOL). The navigation performance requirements are determined by the flight phase, which contains en route, terminal, and approach. The Category-I (CAT-I) is a precision approach which needs reliable lateral and vertical guidance.

With the evolution of GNSS, there will be more navigation satellites broadcasting signals in two or more sub-bands in the aeronautical radio navigation satellite service (ARNSS) band. Using dual-frequency GNSS measurements, it is able to eliminate the ionospheric delay, which will greatly enhance the integrity and positioning accuracy and make it possible to achieve the precision approach relying on GNSS.

Receiver autonomous integrity monitoring (RAIM) is widely used to monitor the integrity performance of navigation systems in civil aviation, urban transport system, and rails [1,4,5,6]. With the evolution of GNSS, the number of available satellites increases greatly, which results in the increasing possibility of simultaneous multiple satellites fault. Although the residual-based RAIM is able to monitor the multiple-satellite fault, it is proved that the detection boundary of the multiple hypothesis solution separation (MHSS) RAIM is closer to the optimal detection boundary than that of residual-based RAIM [7]. Advanced RAIM (ARAIM) is the next generation of RAIM, which is proposed by EU-U.S. Cooperation on Satellite Navigation Working Group C, and it is based on MHSS [8,9,10]. ARAIM is adopted as the major method by the GNSS Evolutionary Architecture Study (GEAS) Working Group [11]. ARAIM will be widely used in the next generation of air traffic management (ATM) systems [12].

As ARAIM is based on MHSS, it needs hundreds of subsets to conduct the fault detection (FD) test and to obtain the integrity bound [13,14]. The sheer number of subsets the ARAIM needs to evaluate makes the current ARAIM too complex to be practically implemented in the airborne navigation system. In recent years, multiple methods were proposed to reduce the computational load of ARAIM. A method using the subset excluding the satellites of the same orbit to evaluate the single satellite fault subsets is presented in [15]. A method with a fixed number of subsets is proposed to reduce the computational load [16]. A method with a feedback structure to obtain the prior probability accumulation which is used to determine the subsets to evaluate is proposed to reduce the number of subsets in [17]. However, all the methods above are for the GNSS alone (without inertial aiding) ARAIM, and the integrity performance is somehow sacrificed. Moreover, they are not always able to meet the stringent integrity requirements of CAT-I precision approach.

The navigation system applied in air transport should also meet the continuity requirements of CAT-I. The continuity risk accounts for all events that lead to interruption of positioning services. Although many factors like clock error, ephemeris error, multipath, and receiver thermal noise, are able to cause positioning services interruption, false alarm is considered as the main factor interrupting the positioning services [18]. To meet the continuity requirements of the precision approach, the navigation system needs to continue providing positioning services after faults being detected. Fault exclusion (FE) is used to find the failing satellites, exclude the failing satellites, and enable the receiver to continue providing positioning services and integrity performance monitoring after exclusion [18]. If the integrity performance after exclusion is able to meet the integrity requirements of the precision approach, the continuity risk is reduced. Multiple fault exclusion algorithms were designed to provide predictive integrity bound after exclusion, but they are for GNSS alone (without inertial aiding) ARAIM [18,19,20,21].

As almost all the aircrafts have high-performance inertial navigation systems (INSs, aviation-grade INSs) on board, the research on the tightly coupled GNSS/INS has attracted extensive attention. In recent years, there are several good ideas provided to solve the information fusion problems, such as sequential Monte Carlo (SMC) method for filtering time-varying information under model uncertainty [22,23], and particle filter for the nonlinear problems [24,25]. However, until now, Extend Kalman Filter-based (EKF-based) tightly coupled GNSS/INS is widely used in civil aviation for its high accuracy performance and high robustness. Therefore, multiple EKF-based GNSS/INS ARAIM algorithms were proposed in recent years [26,27]. However, the current GNSS/INS ARAIM is fundamentally based on baseline ARAIM and it still needs to monitor hundreds of subsets to conduct the fault detection test and to obtain integrity monitoring, which results in a huge computational load.

In this paper, a subset-reduced GNSS/INS ARAIM is presented. By using the subset excluding entire constellation to evaluate the single satellite fault subsets and the simultaneous multiple satellites fault subsets, the proposed algorithm can greatly reduce the subsets. Furthermore, to improve continuity performance, fault exclusion for the proposed algorithm is also presented. By using the a priori state estimate of EKF, the proposed fault exclusion test is able to find the failing satellite exactly.

The remaining part of this paper is organized as follows. In the second section, the current ARAIM and the number of subsets it needs to evaluate is delivered. In the third part, the subset-reduced method for the GNSS/INS ARAIM is proposed. In the fourth section, the FD test and the method to obtain the integrity bound of FD only GNSS/INS ARAIM are presented. In the fifth part, the fault exclusion method for the subset-reduced GNSS/INS ARAIM algorithm is presented. To conclude, the synthetic results of the proposed method are presented.

## 2. Number of Subsets of the Current ARAIM

The number of the subsets is determined by the prior probabilities of satellite fault and constellation fault which are provided in the integrity support message (ISM) [10]. Further, the ARAIM proposed a probability threshold, $p_{thre},$ to bound the integrity risk (IR) coming from the unmonitored satellite fault subsets and unmonitored constellation fault subsets, and it is also presented in the ISM [8]. According to the number of failing satellites the subset contained, the fault subsets can be divided into multiple fault groups [28]. The zeroth-order fault group doesn’t have the failing satellite and it is a fault free subset. Further, the first-order fault mode group contains single satellite fault subsets and single constellation fault subsets. In addition, the second-order fault group contains the simultaneous two satellites fault subsets, and one-satellite and one-constellation fault subsets. The probability of the group is the sum of the probabilities of all fault subsets in this group. Furthermore, if the sum of the probabilities of the group (e.g., first-order group) and its higher-order groups (e.g., second-order group, third-order group, and so on) is smaller than the $p_{thre},$ all the fault subsets in this group and its higher-order group need no monitoring. Otherwise, all the fault subsets in this group need to be monitored.

We will denote the prior probability of satellite i fault and the prior probability of constellation i fault as $p_{sat,i}$ and $p_{const,i},$ respectively. The prior probability of fault free subset (zeroth-order group) is given by [29]:

(1) $$p_{rm,0}=\overset{N_{const}}{\underset{j=1}{\prod }}\left(1-p_{const,j}\right)\;\overset{N_{sat}}{\underset{i=1}{\prod }}\left(1-p_{sat,i}\right)$$

where $N_{sat}$ is the number of available satellites, $N_{const}$ represents the number of available constellations. The sum of the probabilities of the first-order group and its higher-order fault mode groups is given by [29]:

(2) $$p_{rm,1}=1-p_{rm,0}$$

Similar to the above formula, the sum of the probabilities of the second-order group and its higher-order groups is given by [29]:

(3) $$p_{rm,2}=1-p_{rm,0}-p_{rm,0}\left(\overset{N_{sat}}{\underset{i=1}{\sum }}\frac{p_{sat,i}}{1-p_{sat,i}}+\overset{N_{const}}{\underset{j=1}{\sum }}\frac{p_{const,j}}{1-p_{const,j}}\right)$$

The total probability of the third-order group and its higher-order groups is given by [29]:

(4) $$\begin{array}{l} p_{rm,3}=1-p_{rm,0}\left(1+\sum_{i=1}^{N_{sat}}\frac{p_{sat,i}}{1-p_{sat,i}}+\sum_{j=1}^{N_{const}}\frac{p_{const,j}}{1-p_{const,j}}\right)-\\ p_{rm,0}\left(\sum_{i_{1}<i_{2}}\frac{p_{sat,i_{1}}}{1-p_{sat,i_{1}}}\frac{p_{sat,i_{2}}}{1-p_{sat,i_{2}}}-\;\sum_{i\notin j}\frac{p_{sat,i}}{1-p_{sat,i}}\frac{p_{cosnt,j}}{1-p_{const,j}}\right) \end{array}$$

To reduce the computational burden, the method to obtain the upper bound of the probability of the group is provided. The probability upper bound of the $r^{th}-order$ group and its higher-order groups is given by [29]:

(5) $$p_{rm,r}\leq \underset{i_{1}<i_{2}<i_{3}<\ldots <i_{N_{sat}},j_{1}<j_{2}\cdots <j_{N_{const}}}{\sum }p_{sat,i_{1}}p_{sat,i_{2}}p_{sat,i_{3}}\cdots p_{sat,i_{N_{sat}}}p_{j_{1}}\ldots p_{j_{const}}$$

If $p_{rm,r}\geq p_{thre}\;$and $p_{rm,r+1}<p_{thre},$ the ARAIM only monitors the subsets in the $r^{th}-order$ group and its lower order groups.

In general, we only need to monitor fault free mode, first-order fault modes, and second-order fault modes. Suppose there are $N_{const}$ constellations, then there are $\left(N_{const}-1\right)N_{sat}$ one-satellite and one-constellation out fault subsets, $N_{sat}$ single satellite faults, and $N_{sat}\left(N_{sat}-1\right)/2$ simultaneous two satellites fault subsets. Then the total number of subsets is given by:

(6) $$\begin{array}{c} N_{t}=N_{const}N_{sat}+N_{sat}\left(N_{sat}-1\right)/2 \end{array}$$

The total number of first-order and second-order subsets is presented in Table 1. The number is obtained based on the Global Positioning System (GPS) and the BeiDou Navigation Satellite System (BDS) two constellations. Table 1 shows that there are hundreds of subsets the current ARAIM needs to evaluate. Meanwhile, the computational load of each subset is almost the same as that of one complete positioning process. Therefore, the computational load of the current ARAIM is so high that it is unpractical for the current ARAIM to applicate in the airborne receiver [30].

## 3. Number of Subsets of the Proposed ARAIM

As the computational load is linear to the number of subsets, reducing the number of subsets is essential for reducing computational load. However, reducing subset always degrades the integrity performance [28]. On account of the integrity requirements of CAT-I being tight for the GNSS alone ARAIM, the integrity performance of the subset-reduced GNSS alone ARAIM is not always able to meet CAT-I requirements. Meanwhile, almost all aircraft have high-performance INS on board. Therefore, a subset-reduced GNSS/INS ARAIM is proposed to reduce the number of subsets while ensuring that the ARAIM is able to meet the requirements of CAT-I. The proposed algorithm is suitable for two-constellation scenarios, three-constellation scenarios, and four-constellation scenarios. For clarity and without loss of generality, the proposed method is presented in two-constellation scenarios involving GPS and BDS. It can be easily extended to other scenarios. For two-constellations scenarios, ARAIM needs to monitor the zeroth-order fault free group, first-order fault mode group, and second-order fault mode group. As the probability of INS fault is far smaller than the probability of satellite faults, the INS fault is neglected.

We denote the state estimate obtained by INS and all-in-view GPS/BDS satellites as ${\hat{x}}_{k,0}.$ WE name the state estimate obtained by the INS and all-in-view GPS as ${\hat{x}}_{k,1},$ which is also named BDS-out fault subset. The state estimate by the INS and all-in-view BDS satellites is named ${\hat{x}}_{k,2},$ which is also named GPS-out fault subset. We denote the a priori position estimate as ${\hat{x}}_{k,3}$ which is also named the GPS-and-BDS-out fault subset. Furthermore, the prior probability of fault free subset is given by:

(7) $$\begin{array}{c} p_{k,0}=\left(1-p_{const,GPS}\right)\left(1-p_{const,BDS}\right)\overset{N_{sat}}{\underset{i=1}{\prod }}\left(1-p_{sat,i}\right) \end{array}$$

where the $p_{const,GPS}\;\mathrm{and}\;p_{const,BDS}$ is the prior probability of GPS constellation fault and BDS constellation fault, respectively. The probability of the BDS-out fault subset is given by:

(8) $$p_{k,1}=p_{const,BDS}+\overset{N_{BDS}}{\underset{i=1}{\sum }}p_{sat,i}$$

where $N_{BDS}$ represents the number of available BDS satellites. In addition, the prior probability of the GPS-out fault subset is given by:

(9) $$p_{k,2}=p_{const,GPS}+\overset{N_{GPS}}{\underset{i=1}{\sum }}p_{sat,i}$$

where $N_{GPS}$ represents the number of available GPS satellites. Furthermore, the prior probability of the GPS-and-BDS-out fault is given by:

(10) $$p_{k,3}=p_{const,GPS}+p_{const,BDS}+\overset{N_{sat}}{\underset{i=1}{\sum }}p_{sat,i}$$

The BDS-out fault subset can evaluate the single BDS out fault modes, simultaneous two individual BDS satellites out fault modes, and BDS constellation out fault mode. The GPS-out fault subset can monitor the single GPS out fault modes, simultaneous two individual GPS satellites out fault modes, and GPS constellation out fault mode. The GPS-and-BDS-out fault subset can evaluate the single BDS satellite and single GPS satellite out fault modes, single BDS out and GPS constellation out fault modes, single GPS satellite out and BDS constellation out fault modes. That means the new four fault subsets are able to monitor the zeroth -order, first-order and second-order subsets of the current ARAIM.

Table 2 presents the comparison of the number of subsets of the current ARAIM and the subset-reduced ARAIM. It is easy to find that the number of subsets of the subset-reduced ARAIM is about 2% of that of the current ARAIM, which reduces the computational burden dramatically.

**Figure 1:**
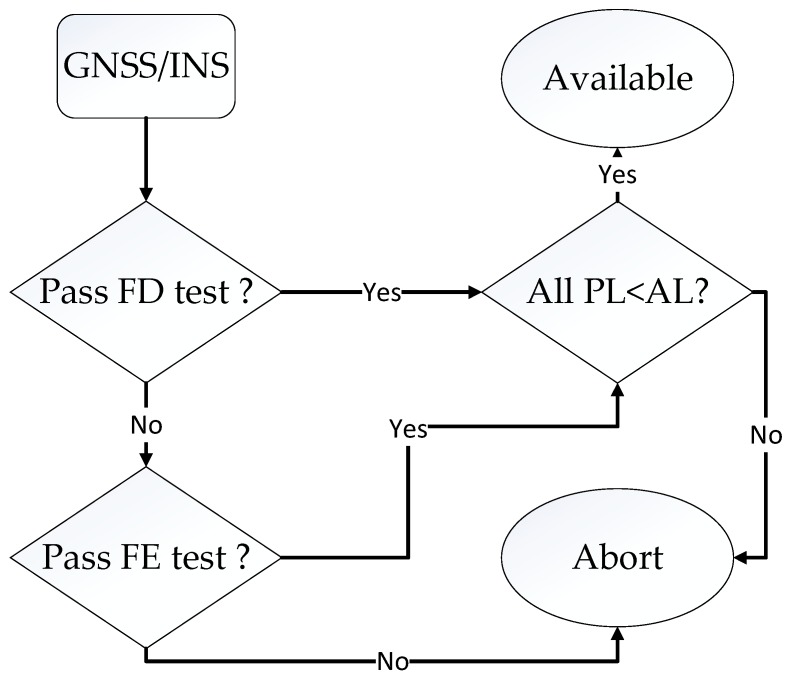
Flow diagram of the fault detection and exclusion process.

The proposed subset-reduced GNSS/INS ARAIM consists of two-part, fault detection and fault exclusion. The overview flow diagram of the ARAIM is presented in Figure 1. The protection level (PL) and alert limit (AL) in this figure represent the protection level and alert limit, respectively. Furthermore, the detailed FD and FE process of the proposed subset reduced ARAIM is presented in the following Section 4 and Section 5, respectively.

## 4. Fault Detection of Subset-Reduced GNSS/INS ARAIM

As shown in Section 3, the subset-reduced ARAIM is also based on MHSS, then the fault detection statistics are the difference between the ${\hat{x}}_{k,0}$ and the ${\hat{x}}_{k,i}\;\left(i=1,2,3\right).$ There a detection test using the pseudorange residuals of the GNSS measurements is added to improve the accuracy of detecting failing satellites. As the continuity risk is mainly from the false alarm [18], the thresholds of the fault detection test are determined by the continuity requirements. As there are two fault detection tests and each of them can cause a false alarm, it is assumed that the continuity risk coming from the two-fault detection test is equal. The continuity risk requirement of CAT-I is $8\times 10^{-6}/15s.$ To be conservative, the continuity risk allocation to the false alarm is set to $4\times 10^{-6}/15s$ [8]. Then half of the false alarm probability, i.e., $2\times 10^{-6}/15s$ is allocated to the MHSS fault detection and the other half is allocated to the pseudorange residual fault detection.

### 4.1. MHSS Fault Detection Test

As the state estimate of last epoch is the same for all the subsets, the MHSS fault detection statistic of the subset i (i=1,2,3) is given by:

(11) $$\begin{array}{ll} FD_{MS,all,k,i} & =\left|{\hat{x}}_{k,i}-{\hat{x}}_{k,0}\right|\\ & =\left|\Delta {\hat{x}}_{k,i}-\Delta {\hat{x}}_{k,0}\right|\\ & =\left|\left(K_{k,i}-K_{k,0}\right)\left(z_{k}-H_{k|k-1}\Delta {\hat{x}}_{k|k-1,0}\right)\right| \end{array}$$

where $K_{k,i}$ is Kalman gain of the subset i at epoch k, and it is given by [31]:

(12) $$K_{k,i}={\hat{P}}_{k|k-1,0}H_{k,i}^{T}{\left(R_{k,i}+H_{k,i}{\hat{P}}_{k|k-1,0}^{T}H_{k,i}^{T}\right)}^{-1}$$

where ${\hat{P}}_{k|k-1,0}$ is the error covariance matrix of a priori state estimate. Further, it is given by [31,32]:

(13) $${\hat{P}}_{k|k-1,0}=\emptyset_{k-1}{\hat{P}}_{k-1,0}\emptyset_{k-1}^{T}+Q_{k}$$

where $\emptyset_{k-1}$ is the state transition matrix which translates the last epoch state estimate to the current epoch a priori state estimate. ${\hat{P}}_{k-1,0}$ is the error covariance matrix of the state estimate of last epoch. In addition, $Q_{k}$ is the covariance matrix of the process noise. $H_{k,0}$ is the geometry matrix which translates the state error to the pseudorange error and pseudorange rate error. $H_{k,i}$ is the geometry matrix of the subset i, and it is obtained by:

(14) $$H_{k,i}=E_{i}H_{k,0}$$

where $E_{i}$ is an identity matrix except that the diagonal elements corresponding to the satellites on the hypothetical fault list of subset i are replaced by zero. $R_{k,i}$ is the covariance matrix of the GNSS measurement errors of subset i. It is obtained by:

(15) $$R_{k,i}=R_{k,0}E_{i}$$

It is easy to obtain the covariance matrix of the fault detection statistics, which is given by:

(16) $$\begin{array}{c} \Omega_{MS,FD,all,k,i}=E\left\{\left(K_{k,i}-K_{k,0}\right)\left(z_{k}-H_{k|k-1}\Delta {\hat{x}}_{k|k-1,0}\right){\left(z_{k}-H_{k|k-1}\Delta {\hat{x}}_{k|k-1,0}\right)}^{T}{\left(K_{k,i}-K_{k,0}\right)}^{T}\right\}\\ =\left(K_{k,0}-K_{k,i}\right)R_{k,0}\left(K_{k,0}^{T}-K_{k,i}^{T}\right)+\left(K_{k,0}-K_{k,i}\right)H_{k|k-1}{\hat{P}}_{k|k-1}H_{k|k-1}^{T}\left(K_{k,0}^{T}-K_{k,i}^{T}\right) \end{array}$$

The fault detection of ARAIM is focused on the position domain [33]. Then a matrix is constructed to extract the position part from the state estimate. The position domain MHSS fault detection statistics and their corresponding covariance matrix are rewritten as:

(17) $$\begin{array}{c} \begin{array}{c} FD_{MS,k,i}=AFD_{MS,all,k,i}\\ \Omega_{MS,FD,k,i}=A\Omega_{FD,all,k,i}A^{T} \end{array} \end{array}$$

where matrix A is used to extract the needed position part from the state estimate, and it is defined by:

(18) $$A=\left[\begin{array}{cccc} 1 & 0 & 0 & \ldots \\ 0 & 1 & 0 & \ldots \\ 0 & 0 & 1 & \ldots \end{array}\right]$$

In addition, the MHSS fault detection threshold of subset i in the q direction is given by:

(19) $$T_{MS,FD,k,i,q}=\kappa_{MS,q}\Omega_{MS,FD,k,i,q}$$

where q=1, 2, and 3, denote the east, north, and vertical components of the threshold, respectively. The $\Omega_{MS,FD,k,i,q}$ is the $q^{th}$ diagonal element of $\Omega_{MS,FD,k,i}.$ Furthermore, the factor $\kappa_{MS,q}$ is defined by:

(20) $$\kappa_{MS,q}=\{\begin{array}{cc} Q^{-1}\left(\frac{p_{FA\_\mathrm{HOR}\_\mathrm{MS}\;}}{4N_{fault}}\right) & \;q=1,2\\ Q^{-1}\left(\frac{p_{FA\_\mathrm{VERT}\_\mathrm{MS}}}{2N_{fault}}\right) & \;q=3 \end{array}$$

where Q is the complement of the zero-mean cumulative standard normal distribution function, and it is defined by:

(21) $$Q\left(u\right)=\frac{1}{\sqrt{2\pi }}\overset{+\infty }{\underset{u}{\int }}e^{-\frac{t^{2}}{2}}dt$$

where $Q^{-1}$ is the inverse function of the above formula. The $p_{FA\_\mathrm{HOR}\_\mathrm{MS}\;}\;$and $p_{FA\_\mathrm{VERT}\_\mathrm{MS}}$ are the false alarm probability allocations to the MHSS fault detection test in the horizontal and vertical direction, respectively.

### 4.2. Residual-Based Fault Detection Test

To improve the accuracy of detecting fault, pseudorange residual fault detection is introduced. The pseudorange residuals are the difference between the pseudorange measurements and the predicted pseudorange based on the state estimate, $\;{\hat{x}}_{k,0}.$ We can extract the position part of ${\hat{x}}_{k,0},$ and name it as ${\hat{x}}_{k,0,pos}.$ Then the pseudorange residuals are given by:

(22) $${ℰ}_{FD,k}=z_{k}-G_{W}{\hat{x}}_{k,0,pos}$$

where $G_{W}$ is the weighted geometry matrix, which is able to translate the position estimate to the pseudorange. ${ℰ}_{FD,k}$ is a $N_{sat}-dimensional$ vector. $z_{k}$ is pseudorange measurement and it can be modelled as:

(23) $$z_{k}=G_{w}{\hat{x}}_{k,0,pos}+G_{w}\Delta {\hat{x}}_{k,0,pos}+n_{k}$$

Substituting Equation (23) into the Equation (22), ${ℰ}_{FD,k}$ can be rewritten as:

(24) $${ℰ}_{FD,k}=G_{w}\Delta {\hat{x}}_{k,0,pos}+n_{k}$$

where $n_{k}$ is the pseudorange noise vector, which is assumed to be a Gaussian white noise sequence. Then covariance matrix of ${ℰ}_{FD,k}$ is obtained by:

(25) $$\Omega_{rd,k}=G_{w}{\hat{P}}_{k,0,pos}G_{w}^{T}+R_{k,0}$$

where $R_{k,0}$ is the covariance matrix of noise $n_{k}.$ ${\hat{P}}_{k,0,pos}$ is the covariance matrix of the position error vector $\Delta {\hat{x}}_{k,0,pos}.$ Then the residual-based fault detection statistic at epoch k is obtained by:

(26) $$FD_{RD,FD,k}={ℰ}_{FD,k}^{T}\Omega_{rd,k}^{-1}{ℰ}_{FD,k}$$

The fault detection statistic is a chi-square distribution with a degree of freedom of $N_{sat}.$ The corresponding fault detection threshold is given by:

(27) $$T_{RD,FD,k}=Q_{Chi}^{-1}\left(p_{FA,RD},N_{sat}\right)$$

where $Q_{Chi}^{-1}$ is the chi-square inverse cumulative distribution function. Further, $p_{FA,RD}$ is the false alarm probability allocations to the residual-based fault detection test.

**Figure 2:**
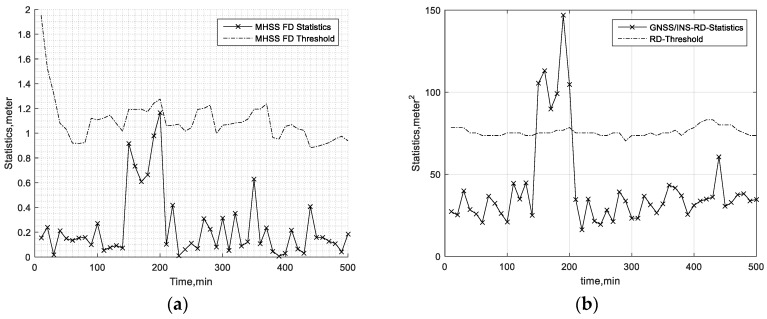
Fault detection (FD) test in the first case. (**a**) Multiple hypothesis solution separation (MHSS) FD test. (**b**) Residual Fault Detection (RD FD) test.

**Figure 3:**
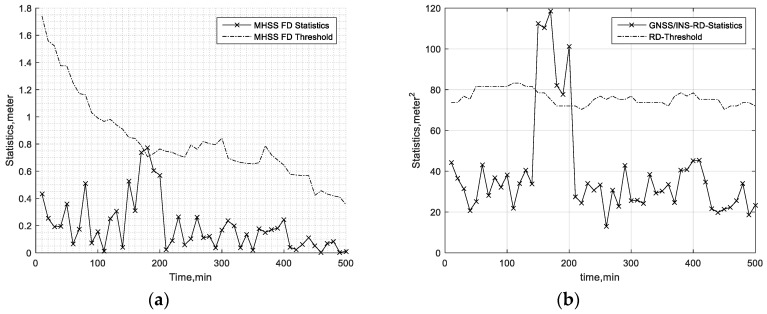
FD test in the second case. (**a**) MHSS FD test. (**b**) RD FD test.

To show the accuracy of the fault detection, FD results in two cases are presented. In the first case, a 20-m bias is added on the pseudorange of the first satellite of the available GPS satellites during the 150th minute to 200th minute period. In the second case, a 15-m bias is added on the third satellite and fifth satellite (randomly selected) of the available GPS satellites during the 150th minute to 200th minute. The other simulation parameters are presented in Table 3. The aviation-grade INS simulation parameters are referred to in [31,34,35]. In addition, the GNSS simulation parameters are referred to in [1,11]. In Table 3, the gyro time constant and the accelerometer bias time constant are the correlation time of the first-order Markov bias of the gyro and accelerometer, respectively. Furthermore, the approach in Table 3 is one of the flight phases, and it is always set to 150 s. The hazardous monitoring information (HMI) and Effective Monitor Threshold-I (EMT-I) are defined in Section 4.3 and Section 6.1, respectively. Figure 2 and Figure 3 present the FD test statistics and the corresponding thresholds in the first and second cases, respectively. The results show that the MHSS FD is not always able to find the pseudorange error. The reason why the MHSS FD can’t detect these pseudorange errors is that this pseudorange error and the dilution of precision of this satellite is so small that they do not cause the position error exceeding the threshold at the current epoch. The results in Figure 2 and Figure 3 also show that the pseudorange residual fault detection test is able to detect this pseudorange bias. The results show that the fault detection test of the proposed ARAIM is able to find the bias accurately.

### 4.3. Integrity Monitoring without Fault Being Detected

If all the fault detection statistics are lower than the corresponding fault detection thresholds, the integrity monitoring is started. The protection level (PL) is the integrity bound at position domain and it is always used to evaluate the integrity performance of the navigation system. The hazardous monitoring information (HMI) probability refers to the probability that position error caused by the undetected fault exceeds the specified alert limit (AL) of CAT-I. Similar to the integrity risk bound computing process of the current ARAIM [8], the bound of the actual integrity risk (IR) in the q direction at epoch k, $p_{FD,HMI,k,q},$ is obtained by:

(28) $$\begin{array}{ll} P_{FD,HMI,k,q} & =\sum_{i=0}^{3}P\left(\begin{array}{c} \left|{\hat{x}}_{k,0,pos,q}-x_{k,q}\right|>PL_{FD,k,q},\\ \left|{\hat{x}}_{k,i,pos,q}-{\hat{x}}_{k,0,pos,q}\right|<T_{MS,FD,k,i,q},\\ FD_{RD,\frac{GNSS}{INS},k}<T_{FD,RD,k} \end{array}|subset\;i\;\right)p_{k,i}\\ & \leq 2Q\left(\frac{PL_{FD,k,q}-b_{nom,k,0,q}}{\sigma_{k,0,q}}\right)+\sum_{i=1}^{3}\begin{array}{c} p_{k,i}Q\left(\frac{PL_{FD,k,q}-T_{MS,FD,k,i,q}-b_{nom,k,i,q}}{\sigma_{k,i,q}}\right) \end{array} \end{array}$$

where $PL_{FD,k,q}$ is the protection level in the q direction at epoch k. The $p_{k,i}$ is the prior probability and it is given by Equation (7) through to (10). Further, q=1, 2, and 3 denotes the east, north, and up components of PL, respectively. In addition, $\sigma_{k,i,q}$ is the q direction component of the $\sigma_{k,i},$ i.e., $q^{th}$ diagonal factor of the $\sigma_{k,i}.$ $\sigma_{k,i}$ is the position part of ${\hat{P}}_{k,i},$ the covariance matrix of ${\hat{x}}_{k,i}.$ It can be obtained by [8]:

(29) $$\begin{array}{c} {\hat{P}}_{k,i}=\left(I-K_{k,i}H_{k,i}\right){\hat{P}}_{k|k-1,0}\\ \sigma_{k,i}^{2}=A{\hat{P}}_{k,i}A^{T} \end{array}$$

where $K_{k,i},$ $H_{k,i},$ and ${\hat{P}}_{k|k-1,0}$ can be obtained by Equations (12)–(14), respectively. $b_{nom,k,i,q}$ is the impact of the nominal bias on the position solution of the subset i. It is obtained by:

(30) $$b_{nom,k,i,q}=K_{k,i,q}b_{nominal,k}$$

where $K_{k,i,q}$ is the $q^{th}$ row of the $K_{k,i}$ which is defined by Equation (12). $b_{nominal,k}$ is the nominal bias which is defined in [10]. $PL_{FD,k,q}$ can be obtained by making the integrity risk bound expressed by Equation (28) equal to the integrity risk threshold. It is expressed as:

(31) $$2Q\left(\frac{PL_{FD,k,q}-b_{nom,k,0,q}}{\sigma_{k,0,q}}\right)+\overset{3}{\underset{i=1}{\sum }}\begin{array}{c} p_{k,i}Q\left(\frac{PL_{FD,k,q}-T_{MS,FD,k,i,q}-b_{nom,k,i,q}}{\sigma_{k,i,q}}\right)= \end{array}p_{HMI,q}\left(1-\frac{p_{not\;monitored}}{p_{HMI}}\right)$$

where the right part of the Equation (31) is the integrity risk threshold allocation to q direction. $p_{HMI}$ represents the total HMI probability requirement. And $p_{HMI,q}$ means the HMI probability allocation to the q direction. $p_{not\;monitored}$ is the HMI probability coming from the subsets that do not need to be monitored [29]. Furthermore, the $PL_{FD,k,q}$ solution of Equation (31) is the FD only (without fault detected) protection level in the q direction.

If the FD only integrity bound, $PL_{FD,k,q},$ is not able to meet the PL requirement of CAT-I, the receiver alerts the user to abort the approach. If any subset fails the FD test, the fault exclusion process is started.

## 5. Fault Exclusion of Subset-Reduced ARAIM

The goal of the fault exclusion test is to identify the failing satellite accurately and to continue providing positioning services after exclusion. Fault exclusion algorithm contains two parts: the fault exclusion test and the integrity performance evaluation after exclusion.

### 5.1. Fault Exclusion Test

The difference between the pseudorange measurements and the pseudorange prediction based on the a priori position estimate ${\hat{x}}_{k,3}$ is used as the fault exclusion statistics. The pseudorange residual of satellite j is given by:

(32) $$Ps_{FE,k,j}=\Gamma_{j}^{T}\left(z_{k}-G_{w}{\hat{x}}_{k,3,pos}\right)$$

where $G_{w}$ is defined in (22). ${\hat{x}}_{k,3,pos}$ is the position part of ${\hat{x}}_{k,3}.$ In addition, $\Gamma_{j}$ is used to extract the $j^{th}$ element of the pseudorange residual vector. $\Gamma_{j}$ is a vector whose elements are all zeros except that the $j^{th}$ element equals one. It is defined by:

(33) $$\Gamma_{j}=\begin{array}{c} 0\\ \vdots \\ \left[1\right]\\ \vdots \\ 0 \end{array}\;$$

Furthermore, the pseudorange $z_{k}$ contains the true distance between the true position and the satellites as well as the pseudorange noise. Given this, it can be modelled as:

(34) $$\begin{array}{ll} z_{k} & =G_{w}x_{k,pos}+n_{k}\\ & =G_{w}{\hat{x}}_{k,3,pos}+G_{w}\left(x_{k,pos}-{\hat{x}}_{k,3,pos}\right)+n_{k}\\ & =G_{w}\Delta {\hat{x}}_{k,3,pos}+G_{w}{\hat{x}}_{k,3,pos}+n_{k} \end{array}$$

where $x_{k,pos}$ is the true position. Substituting (34) into (32), the fault exclusion statistic of the satellite j is obtained by:

(35) $${ℰ}_{FE,k,j}=\frac{\Gamma_{j}^{T}\left(G_{W}\Delta {\hat{x}}_{k,3,pos}+n_{k}\right)}{\Omega_{FE,k,j}}$$

where $\Omega_{FE,k,j}$ is the standard deviation of $Ps_{FE,k,j}.$ Substituting Equation (34) into Equation (32), then $\Omega_{FE,k,j}\;$is obtained by:

(36) $$\Omega_{FE,k,j}=\sqrt{E\left\{Ps_{FE,k,j}Ps_{FE,k,j}^{T}\right\}}=\sqrt{\Gamma_{j}^{T}\left(G_{w}{\hat{P}}_{k|k-1,3,pos}\;G_{w}^{T}+R_{k}\right)\Gamma_{j}}$$

where ${\hat{P}}_{k|k-1,3,pos}$ is the position part of the covariance matrix of the a priori state estimate of the subset three. It is easy to get that ${ℰ}_{FE,k,j}$ is normal Gaussian white noise. Then the threshold of the fault exclusion statistic of the satellite j is obtained by:

(37) $$T_{\mathrm{FE},k,j}=Q^{-1}\left(p_{\mathrm{FE}}/N_{\mathrm{sat}},0,1\right)$$

where Q is defined in Equation (21) and $p_{FE}$ is the probability of wrong exclusion. If the subset exceeding the FD threshold contains the satellite failing to pass the FE test, the receiver will continue providing the navigation services after excluding the identified satellite. Otherwise, the receiver alerts the user to abort the approach.

**Figure 4:**
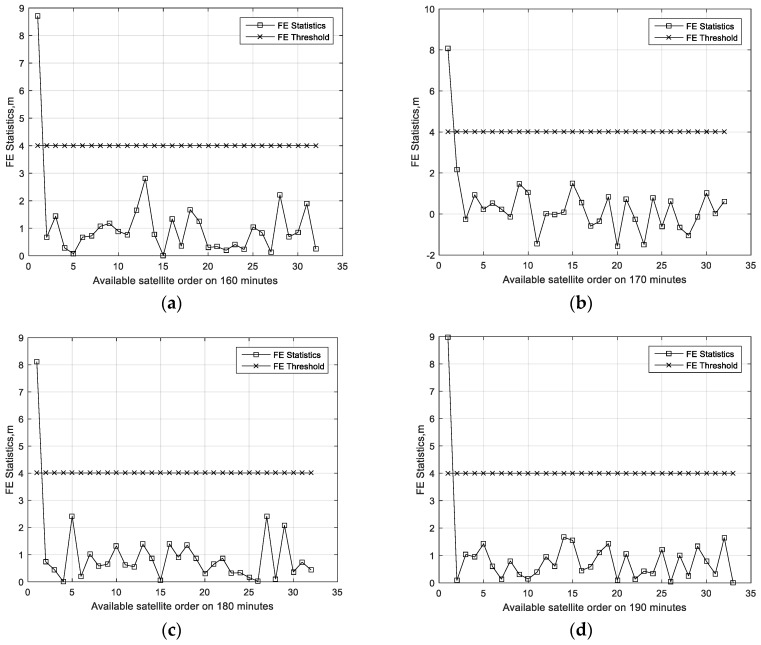
Fault exclusion (FE) test results of the subset reduced GNSS/INS ARAIM in the first case. (**a**) FE test on 160 min, (**b**) FE test on 170 min, (**c**) FE test on 180 min, and (**d**) FE test on 190 min.

**Figure 5:**
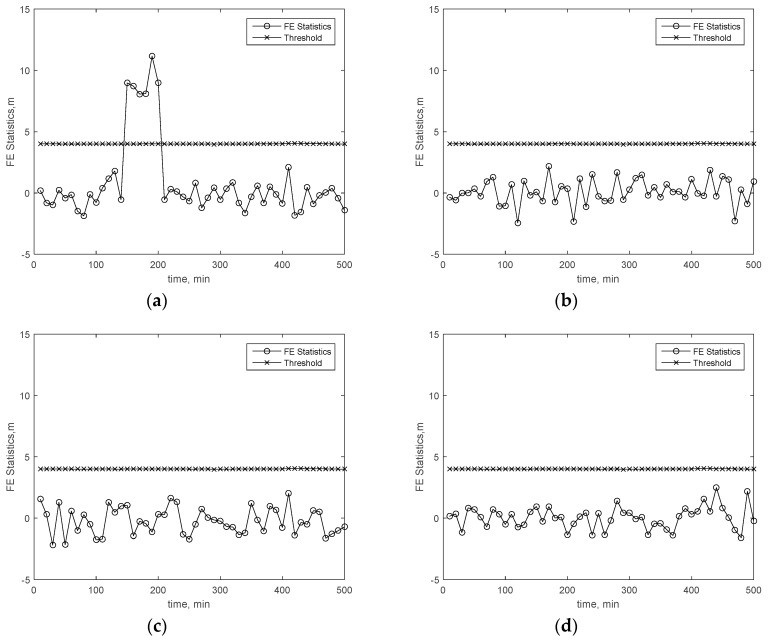
FE test results over all time in the first case. (**a**) FE test of the first satellite, (**b**) FE test of the second satellite, (**c**) FE test of the third satellite, and (**d**) FE test of the fourth satellite.

**Figure 6:**
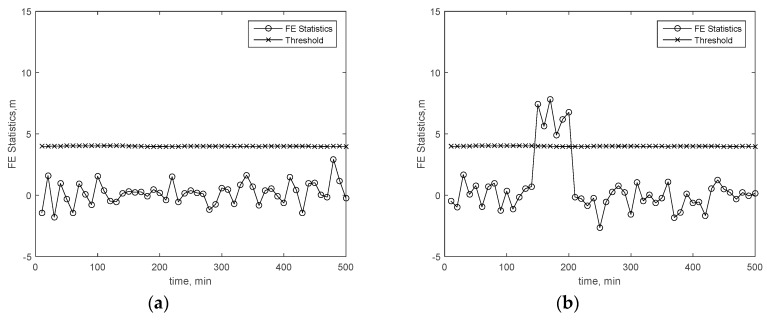

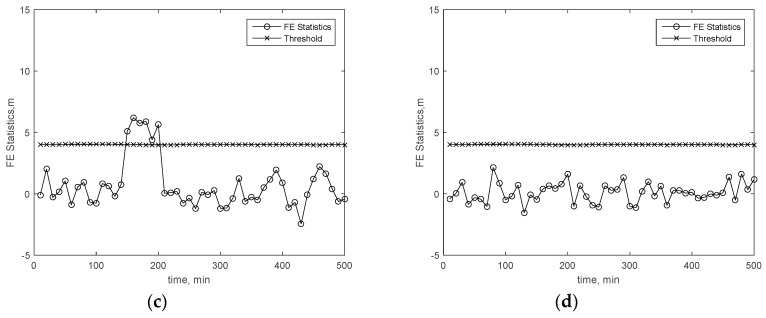
FE test results over all time in the second case. (**a**) FE test of the first satellite. (**b**) FE test of the third satellite (failing satellite). (**c**) FE test of the fifth satellite (failing satellite). (**d**) FE test of the sixth satellite.

Figure 4, Figure 5 and Figure 6 present the FE results of the proposed FE method in the cases stated in Section 4.2. Figure 4a–d present the FE test results of all available satellites within the 160th minute through to the 190th minute in the first case. From the results, it is easy to find that the statistics exceeding the threshold are always coming from the first GPS satellite which the bias is added on. Figure 5a–d present the FE test results in the first case over all time. From these results, it is easy to find that the FE test is able to find the failing satellite exactly. Figure 6a–d present the FE test results in the second case over all time. All the failing-satellite FE statistics exceed the corresponding threshold, which means the FE test is able to find the failing satellites exactly in the second case. That means the FE test is always able to find the failing satellite accurately.

### 5.2. Protection Level Computing after Fault Exclusion

If the excluded satellites are just supposed as not in view and use (28) to obtain the IR upper bound of the rest satellites without the excluded satellite, the actual IR may be beyond the IR estimate [18,36]. Although the probability of excluding normal satellite is very low, it does exist. If the normal satellite is excluded and we use (28) to evaluate IR, the probability of the rest satellite faults will be underestimated [36]. In order to ensure that the IR estimate is able to bound the actual IR, the wrong exclusion must be considered. We denote the right exclusion probability as $p_{rex}$ and the wrong exclusion probability as $p_{wex}.$ The wrong exclusion probability is set to 0.001 in [37]. In order to be conservative, the wrong exclusion probability is set to $p_{wex}=0.01$ here. Furthermore, when the wrong exclusion occurs, the probability of the rest satellite faults is set to 100%. In this way, the IR estimate is able to bound the actual IR after exclusion and then the PL is able to bound the position error.

For the right exclusion, the prior probability of the fault subset i is given by:

(38) $$p_{af,k,re,i}=p_{k,i}$$

where $p_{k,i}$ is defined by Equations (7)–(10) without the identified failing satellite. Further, for the wrong exclusion group, the prior probability of the fault subset i is set to:

(39) $$p_{af,k,we,i}=1$$

The actual IR after exclusion comes from both the wrong exclusion group and the right exclusion group, and it can be expressed as:

(40) $$p_{HMI,k,af,q}=p_{rex}p_{HMI,k,re,q}+p_{wex}p_{HMI,k,we,q}$$

where $p_{HMI,k,re,q}\;\mathrm{and}\;p_{HMI,k,we,q}$ are the integrity risk from the right exclusion and wrong exclusion, respectively. For the right exclusion, the failing satellite is considered as an outage. As stated above, the IR after right exclusion is the probability that after the failing satellite is identified and excluded and the rest subsets pass the FD test, but the position errors still exceed the PL. Similar to the IR bound in the current ARAIM [29], the IR in the q direction at epoch k can be bounded by:

(41) $$\begin{array}{ll} p_{HMI,k,re,q} & =\sum_{i=0}^{i=3}p_{af,k,re,i}\;P\left(\begin{array}{c} \left|x_{k,q}-{\hat{x}}_{af,k,0,pos,q}\right|>PL_{af,k,q},\\ \left|{\hat{x}}_{af,k,i,pos,q}-{\hat{x}}_{af,k,0,pos,q}\right|<T_{af,MS,FD,k,i,q} \end{array}|subset\;i\;\right)\\ & \leq 2Q\left(\frac{PL_{af,k,q}-b_{nom,k,0,q}}{\sigma_{af,k,0,q}}\right)+\sum_{i=1}^{3}p_{af,k,re,i}Q\left(\frac{PL_{af,k,q}-T_{af,MS,FD,k,i,q}-b_{nom,k,i,q}}{\sigma_{af,k,i,q}}\right) \end{array}$$

where $x_{k,q}$ is the true position in the q direction at epoch k. $PL_{af,k,q}$ is the protection level in the q direction after exclusion. ${\hat{x}}_{af,k,i,pos,q}$ is the $q^{th}$ component of the position estimate of subset i (i=0,1,2,3) at epoch k after exclusion. $T_{af,MS,FD,k,i,q}$ is the MHSS detection threshold of subset i in the q direction after exclusion and it can be obtained by Equation (19) without the identified failing satellites. $\sigma_{af,k,i,q}$ is the standard deviation of ${\hat{x}}_{af,k,i,pos,q}$ and it can be obtained by Equation (29) without the identified failing satellite. $b_{af,nom,k,i,q}$ is the impact of the nominal bias on position solution of the subset i after exclusion. It can be obtained by Equation (30) without the identified failing satellites. For the wrong exclusion, the probability of the rest subsets is set to 100%. Similar to the HMI bound of the current ARAIM [29], the HMI probability from the wrong exclusion group can be bounded by:

(42) $$p_{HMI,k,we,q}\leq 2Q\left(\frac{PL_{af,k,q}-b_{nom,k,0,q}}{\sigma_{k,0,q}}\right)+\overset{3}{\underset{i=1}{\sum }}Q\left(\frac{PL_{af,k,q}-T_{af,MS,FD,k,i,q}-b_{nom,k,i,q}}{\sigma_{k,i,q}}\right)$$

As expressed by the Equation (40), the actual IR in q direction at epoch k comes from the right exclusion and wrong exclusion. Substituting Equations (41) and (42) into Equation (40), and the after-exclusion integrity risk bound, $pb_{HMI,k,af,q},$ can be obtained by:

(43) $$\begin{array}{l} p_{HMI,k,af,q}\leq pb_{HMI,k,af,q}\\ =2Q\left(\frac{PL_{af,k,q}-b_{nom,k,0,q}}{\sigma_{k,0,q}}\right)+\sum_{i=1}^{3}\left(p_{af,k,re,i}+1\right)Q\left(\frac{PL_{af,k,q}-T_{af,MS,FD,k,i,q}-b_{nom,k,i,q}}{\sigma_{k,i,q}}\right) \end{array}$$

Similar to the current ARAIM, the PL can be obtained by making the integrity risk bound equal to the CAT-I integrity risk probability allocation to the q direction. Then we have the following:

(44) $$\begin{array}{c} pb_{HMI,k,af,q}=2Q\left(\frac{PL_{af,k,q}-b_{nom,k,0,q}}{\sigma_{k,0,q}}\right)+\sum_{i=1}^{3}\left(p_{af,k,re,i}+1\right)Q\left(\frac{PL_{af,k,q}-T_{af,MS,FD,k,i,q}-b_{nom,k,i,q}}{\sigma_{k,i,q}}\right)\\ =p_{HMI,q}\left(1-\frac{p_{not\;monitored}}{p_{HMI}}\right)\# \end{array}.\;$$

The PL in the q direction after exclusion is the $PL_{af,k,q}$ solution of the Equation (44). If all of the $PL_{af,k,q}\;\left(q=1,2,3\right)$ are smaller than the corresponding requirements of CAT-I, the ARAIM claims the position estimate ${\hat{x}}_{af,k,0,pos}$ available, otherwise, the ARAIM warns the user to abort the approach.

## 6. Performance Estimation

The CAT-I is a precision approach with decision height (DH) not lower than 60 m (200 feet) and runway visual range (RVR) not less than 550m [1]. The purpose of the proposed ARAIM is to achieve the CAT-I precision approach. Navigation performance requirements of CAT-I are presented as follows [1,38]:

1. The probability requirement of hazardously misleading information (HMI), $\;2\times 10^{-7}/\mathrm{approach}\;$;
2. The vertical protection level (VPL) and horizontal protection level (HPL) must be less than 10−15 m and 40 m, respectively;
3. The threshold of continuity risk, $8\times 10^{-6}/15s\;$
4. The 95% vertical accuracy and horizontal accuracy should be lower than 4−6 m and 16 m, respectively;
5. The availability requirement is 0.99-0.99999.

### 6.1. EMT-I Computing

A lot of operational trials have shown that the vertical position error larger than 15 m significantly increases the workload of the flight crew and reduces the safety margin greatly [1]. Furthermore, the Federal Aviation Administration (FAA) introduces a concept, i.e., the effective monitor threshold (EMT), in the requirements of LPV-200 to bound the probability of vertical position error larger than 15 m [11]. The ICAO also stipulates that the probability of a position error exceeding 15 m should be lower than $10^{-5}/approach$.

To bound the probability of the vertical error exceeding 15 m, we denote the vertical integrity performance of the subsets with prior probability larger than $10^{-5}/approach$ as EMT-I and its threshold is set to 15 m.

The EMT-I only monitors the subsets with prior probability greater than $p_{EMT-I},$ i.e., the $10^{-5}/\mathrm{approach}.$ Then if there is no fault detected, the EMT-I is the integrity performance of the subsets with the prior probability exceeding $P_{EMT-I}.$ It can be obtained by:

(45) $$\begin{array}{c} EMTi_{FD,k}=\underset{i|p_{fault,i}\rangle p_{EMT-I}}{max}\left(T_{MS,FD,k,i,3}+K_{md,EMT-I,i}\sigma_{k,i,3}\right)\\ K_{md,EMT-I,i}=Q^{-1}\left(\frac{p_{EMT-I}}{p_{k,i}}\right) \end{array}$$

where $T_{MS,FD,k,i,3}$ and $\sigma_{k,i,3}$ are defined in Equations (19) and (29), respectively. In addition, for the scenario after fault exclusion, the EMT-I should monitor the fault modes with $p_{rex}p_{af,k,re,i}$ or $p_{wex}p_{af,k,we,i}$ greater than the $P_{EMT-I}.$ Further, the EMT-I in the q direction after exclusion can be obtained by:

(46) $$\begin{array}{c} EMTi_{af,k}=\underset{i|p_{rex}p_{af,k,re,i}+p_{wex}\rangle p_{EMT-I}}{max}\left(T_{af,MS,FD,k,i,3}+K_{af,md,EMT-I,i}\sigma_{af,k,i,3}\right)\\ K_{af,md,EMT-I,i}=Q^{-1}\left(\frac{p_{EMT-I}}{p_{rex}p_{af,k,re,i}+\;p_{wex}p_{af,k,we,i}}\right) \end{array}$$

If the $EMTi_{FD,k}$ or the $EMTi_{af,k}$ are smaller than the threshold, (15 m), the ARAIM passes the EMT-I test. Otherwise, the ARAIM warns the user to abort the approach.

### 6.2. Integrity Performance

In terms of GNSS navigation, the vertical integrity requirements of CAT-I are more stringent than the horizontal integrity requirements, therefore, we only show the integrity monitoring results in the vertical direction.

The aviation-grade INS simulation conditions and GNSS simulation parameters are presented in Table 3 in Section 4.2. The standard deviation model used here is defined in [8]. In addition, the user range accuracy (URA) [39,40] for integrity is the same as that in the broadcast ephemeris of GPS, 2.4 m.

**Figure 7:**
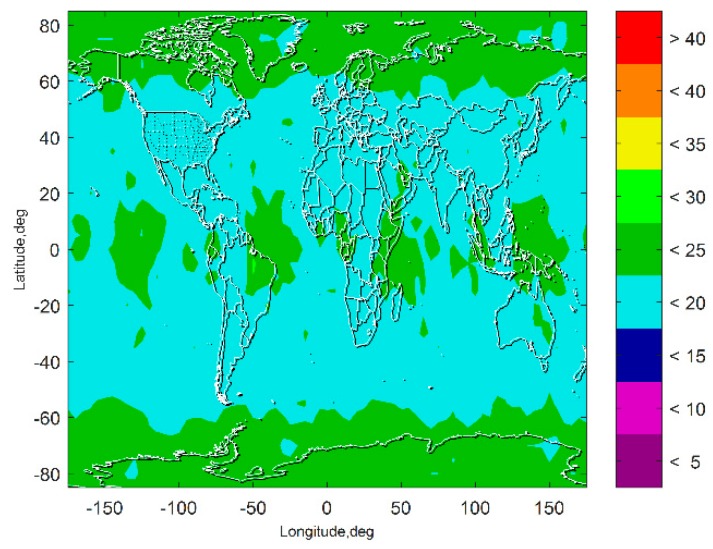
Vertical protection level (VPL) of the subset reduced GNSS alone FD only ARAIM.

**Figure 8:**
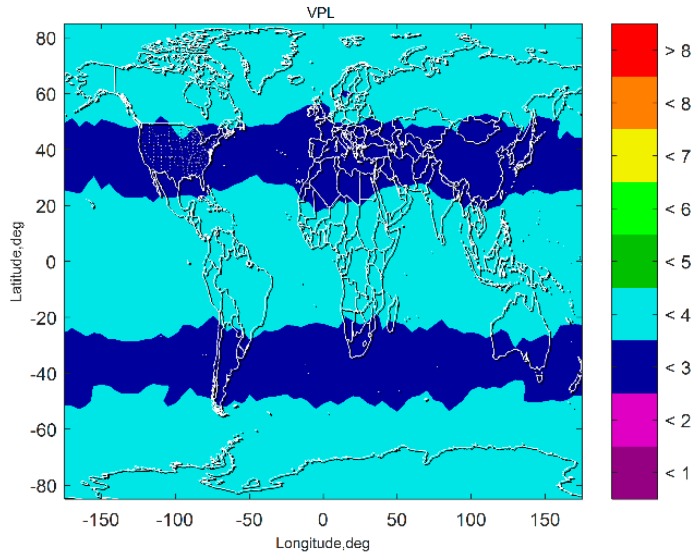
VPL of the subset reduced GNSS/INS FD only ARAIM.

Figure 7 and Figure 8 show the VPL results of the subset-reduced GNSS alone FD only (without INS aiding and without fault excluded) ARAIM and the subset-reduced GNSS/INS FD only ARAIM, respectively. The simulation results show that without the INS aiding, the subset-reduced ARAIM cannot meet the VPL requirement of CAT-I, 10~15 m. Further, the VPL of the proposed GNSS/INS ARAIM is far lower than 15 m. This means the subset-reduced GNSS/INS FD only ARAIM can meet the CAT-I integrity requirements.

**Figure 9:**
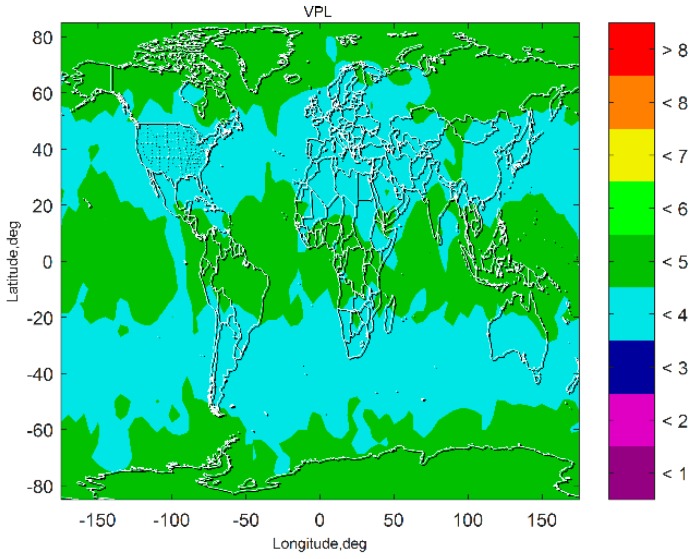
VPL of the subset-reduced GNSS/INS ARAIM after exclusion.

Figure 9 presents the VPL of the subset-reduced GNSS/INS ARAIM after exclusion. The results show that the fault exclusion leads to deterioration of PL performance about one meter. The results also show that although the VPL after exclusion is a little worse than the VPL without fault exclusion, it is still far lower than the PL requirement of the CAT-I. The VPL after exclusion being able to meet the CAT-I integrity requirements means that the fault exclusion for the subset-reduced GNSS/INS ARAIM is able to improve the continuity and availability performance of the navigation system.

**Figure 10:**
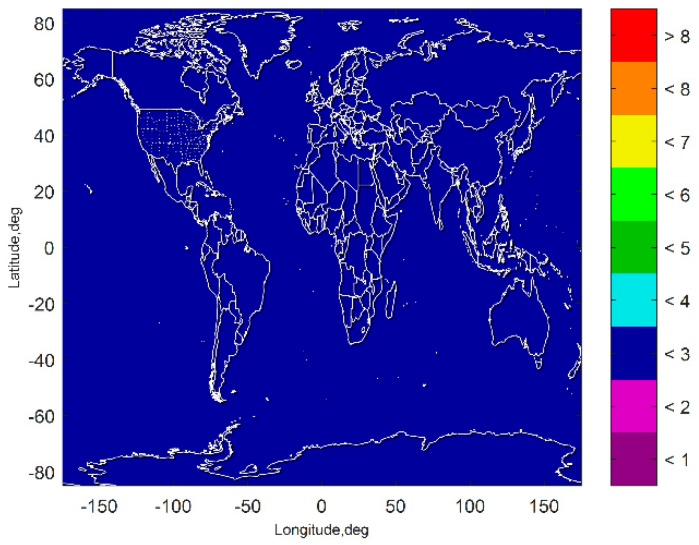
EMT-I of subset-reduced GNSS/INS ARAIM.

**Figure 11:**
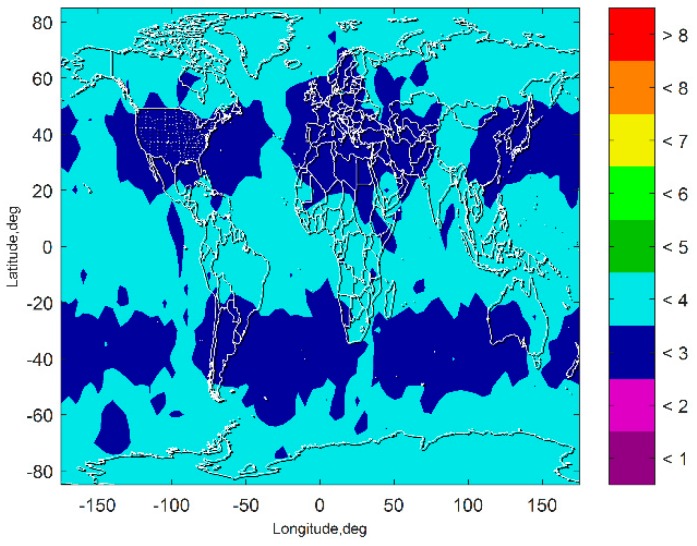
EMT-I of the subset-reduced GNSS/INS ARAIM after exclusion.

Figure 10 and Figure 11 present the EMT-I results of the subset-reduced GNSS/INS FD only ARAIM and the subset-reduced GNSS/INS ARAIM after exclusion, respectively. The results show that although the EMT-I after exclusion is a little worse than the FD only EMT-I, both of them are lower than the 15-m threshold. This means the EMT-I of the ARAIM is also able to meet the requirement.

The results in Figure 7, Figure 8, Figure 9, Figure 10 and Figure 11 show that the subset-reduced ARAIM is still able to meet the requirements of CAT-I with global coverage. This means the proposed method is able to reduce computational load greatly while being able to provide performance meeting CAT-I requirements.

## 7. Conclusions

In this paper, the subset reduced GNSS/INS ARAIM was proposed to reduce the computational load of the current ARAIM. The fault detection test and fault exclusion test of the proposed ARAIM were provided. Moreover, the method to obtain the FD only integrity bound, and after-exclusion integrity bound was presented.

The computational load of the proposed ARAIM was about 2% of that of the current ARAIM, which make it easy for an ARAIM application in the airborne navigation system. Meanwhile, simulation results showed that both the FD only integrity performance and integrity performance after exclusion were able to meet the integrity requirements of CAT-I.

Future research will be focused on further reducing the computational complexity of ARAIM.

## Figures and Tables

**Table 1:** Number of subsets of current advanced receiver autonomous integrity monitoring (ARAIM).

**Number of Satellites**	20	30	40
**Number of Subsets**	210	465	820

**Table 2:** Number of subsets.

	**Constellations**	2		3		4	
		Constellations		Constellations		Constellations	
	**Satellites**	16	24	24	32	32	48
		Satellites	Satellites	Satellites	Satellites	Satellites	Satellites
**Subsets**	**Current ARAIM**	152	326	351	595	628	1324
**Algorithms**	**Subset-Reduced ARAIM**	4	4	7	7	11	11

**Table 3:** Inertial navigation system (INS) and global navigation satellite system (GNSS) simulation conditions.

Parameter	Value	Unit
Gyro angle random walk	0.001	$\;deg/\sqrt{hour}\;$
Gyro bias error	0.01	deg/hour
Gyro time constant	3600	*s*
Accelerometer white noise	$\;10^{-5}\;$	$\;m/s^{2}\;$
Accelerometer bias error	$\;10^{-5}\;$	$\;m/s^{2}\;$
Accelerometer bias time constant	3600	*s*
Probability of satellite fault	$\;10^{-5}\;$	1/hour/Sat
Probability of constellation fault	$\;10^{-4}\;$	1/hour/Const
Hazardous monitoring information (HMI) probability	$\;2\times 10^{-7}\;$	1/approach
Probability of false alert	$\;4\times 10^{-6}\;$	1/15s
Probability of EMT-I	$\;1\times 10^{-5}\;$	1/approach
